# Study Assessing the Quality of Quantification of Estrogen Receptor Protein Expression by Immunohistochemistry and Gene Expression in Breast Cancer

**DOI:** 10.1155/2014/372653

**Published:** 2014-03-06

**Authors:** Sas Leen, Van Laere Steven, Dierick Anne Marie, Duwel Valérie, De Pauw Annemie, Van Den Eynden Gert, Van Dam Peter, Dirix Luc, Vermeulen Peter, Lardon Filip

**Affiliations:** ^1^Translational Cancer Research Unit, GZA Hospitals Sint-Augustinus, Oosterveldlaan 24, 2610 Antwerp, Belgium; ^2^Department of Medical Oncology, University of Antwerp, Universiteitsplein 1, 2610 Antwerp, Belgium; ^3^Department of Oncology, KU Leuven, Herestraat 49, 3000 Leuven, Belgium; ^4^Department of Pathology, AZ Klina Hospital, Augustijnslei 100, 2930 Brasschaat, Belgium; ^5^Department of Pathology, AZ Nikolaas Hospital, Moerlandstraat 1, 9100 Sint-Niklaas, Belgium

## Abstract

Although immunohistochemistry (IHC) is a widely used technique to classify tumors in ER-positive versus ER-negative ones, interlab variabilities can occur. This study aims to investigate the influences of preanalytical and analytical factors on IHC results. For this purpose, the different steps of the preparation of IHC sections and scoring procedures were compared between two participating laboratories and a central lab. There was a significant positive correlation between the IHC results of the participating laboratories and those of the central lab (correlation coefficient > 0.600; * P<0.05*). Nevertheless, some discordant cases for immunostaining (5.3% for ER and 5.6% for PR) and for scoring (10.5% for PR) occur at site 1. Comparing IHC results with ESR1 gene expression results revealed a significant positive correlation (correlation coefficients > 0.769; * P<0.05*). PCR results of ER target genes showed some heterogeneity in the ER-signalling pathway. These results suggest that differences in the IHC procedure between these laboratories did not have a big influence on the end result. Nevertheless, discordant cases caused by preanalytical and analytical lab-specific procedures have been identified.

## 1. Introduction

Breast cancer is one of the most important causes of mortality in women. Intensive research has revealed the existence of 2 nuclear receptors, the estrogen receptor (ER) and progesterone receptor (PR), which play a role in normal development of the breast as well as breast cancer. Generally, ER-positive tumors are treated with endocrine therapy, such as antiestrogens and aromatase inhibitors [1–3]. ER-negative tumors are usually treated with cytotoxic chemotherapy. The first goal in the treatment of patients with breast cancer is to select the most appropriate therapy. For this purpose, patients are screened for the presence of ER and PR in the tumor tissue. In general, ER-positive/PR-positive tumors have the best outcome, while the prognosis of patients with ER-positive/PR-negative tumors is less favorable. Patients with ER-negative/PR-negative tumors have the least favorable prognosis [4]. At the moment, the common technique to determine the status of ER and PR is by immunohistochemistry (IHC) on sections of formalin-fixed and paraffin-embedded tissues (FFPET) followed by scoring of nuclear immunostaining. However, variability between different laboratories occurs, leading to different interpretations and results, with misclassification as a consequence [5]. This variability can be caused by preanalytic differences, such as fixation and protocol variants, as well as analytic differences, such as inter-/intraobserver variability, different scoring systems, and related cut-off values for ER-positivity. The purpose of this study is to assess the quality of (semi)quantification of the ER*α*- and PR-protein expression by IHC on breast adenocarcinoma tissue sections (FFPET) in different pathology laboratories.

## 2. Material and Methods

**Figure 1:**
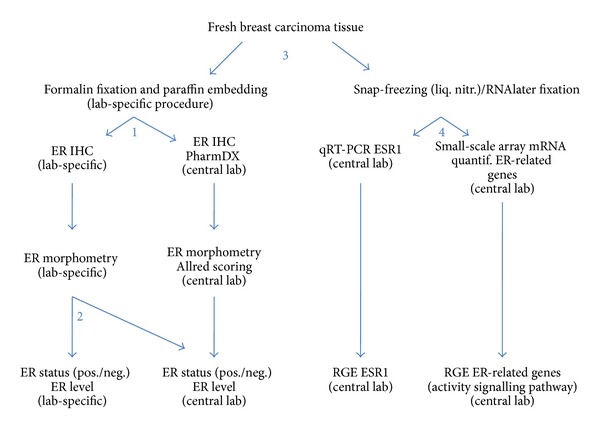
Schematic representation of the study: the scheme indicates the different steps of the study to evaluate the influence of (1) immunostaining; (2) scoring; (3) tissue fixation; and (4) heterogeneity within groups of carcinomas with comparable immunohistochemical receptor status.

The study is a multicenter study wherein 2 clinical sites and translational cancer research unit, the central lab, participate. A schematic representation of the study is given in Figure 1.

### 2.1. Patient Inclusion Criteria

Samples used in this experiment are of patients who gave written approval. The samples include histologically proven breast adenocarcinoma (primary or metastatic, with or without prior treatment), which were at least 1 cm (T1c or higher). The tumors had to be large enough to be able to collect RNAlater fixed carcinoma tissue without influence on the standard diagnostic procedure.

### 2.2. Sample Preparation

After mastectomy or tumorectomy the specimens were transported without delay to the pathology lab. The pathologist took a representative part of the carcinoma (peripheral, viable, and without adjacent normal tissue) immediately upon arrival of the specimen in the lab. The carcinoma tissue was divided into small parts of 5 × 5 × 5 mm or smaller and was submerged in RNAlater solution and incubated at −80°C. Total warm ischemia time did not exceed 20 minutes. Each participating center collected 20 breast cancer samples and sent a referral of each tissue sample and an immunostained section to the central lab.

### 2.3. Immunohistochemistry

Each of the participating laboratories performed an immunohistochemical staining for ER and PR on 20 FFPE tissue sections using their own staining procedure. Next, the IHC coupes were scored by the laboratories themselves using their proper scoring system and rescored by the central lab. At the central lab scoring of the stained FFPE was performed using the Allred histomorphometrical scoring system for ER and PR, resulting in scores (intensity + proportion) from 0 to 8. Scores higher than 2 indicate a positive status [6, 7].

Besides the 20 stained tissue sections, each lab also provided 20 unstained sections that were stained at the central lab using the ER/PR pharmDx kit (DAKO). This kit consists of a cocktail of two mouse monoclonal antibodies for ER, 1D5, and ER-2-123, which bind to different regions of the protein. Finally the stained sections were scored by the researcher and a trained pathologist.

### 2.4. Polymerase Chain Reaction (PCR)

RNA was isolated from the 40 fixed (RNAlater) breast carcinoma tissues received from the participating labs using the RNeasy mini kit (Qiagen, Venlo, Belgium). The amount of RNA was measured by means of the Nanodrop ND3300 Fluorospectrometer (Isogen, Temse, Belgium). Next, 1 µg of RNA was converted to cDNA using the high capacity RNA-to-cDNA kit (Applied Biosystems, Ghent, Belgium). Further, qRT-PCR was done for the ER-coding gene, ESR1 and 11 ER target genes, ESR2, PGR, TGF*β*3, HSD17*β*4, RAB31, STARD10, XBP1, GATA3, MYB, MUC1, and BTG2 (Assays-On-Demand, Applied Biosystems, Ghent, Belgium). 18S and beta-actin (Applied Biosystems, Ghent, Belgium) and Universal Human reference RNA (Stratagene) were used as housekeeping genes and calibrator. The results obtained from qRT-PCR were compared with those of IHC.

### 2.5. Statistical Analysis

The Spearman rank test was used to evaluate correlations between results. Statistical significance was defined at the level of *P* < 0.05. Per participating site, a principal component analysis was performed on the ER target gene expression data to evaluate heterogeneity within the tumors.

## 3. Results

### 3.1. Formalin Fixation and Paraffin Embedding Did Not Influence IHC Results

**Figure 2:**
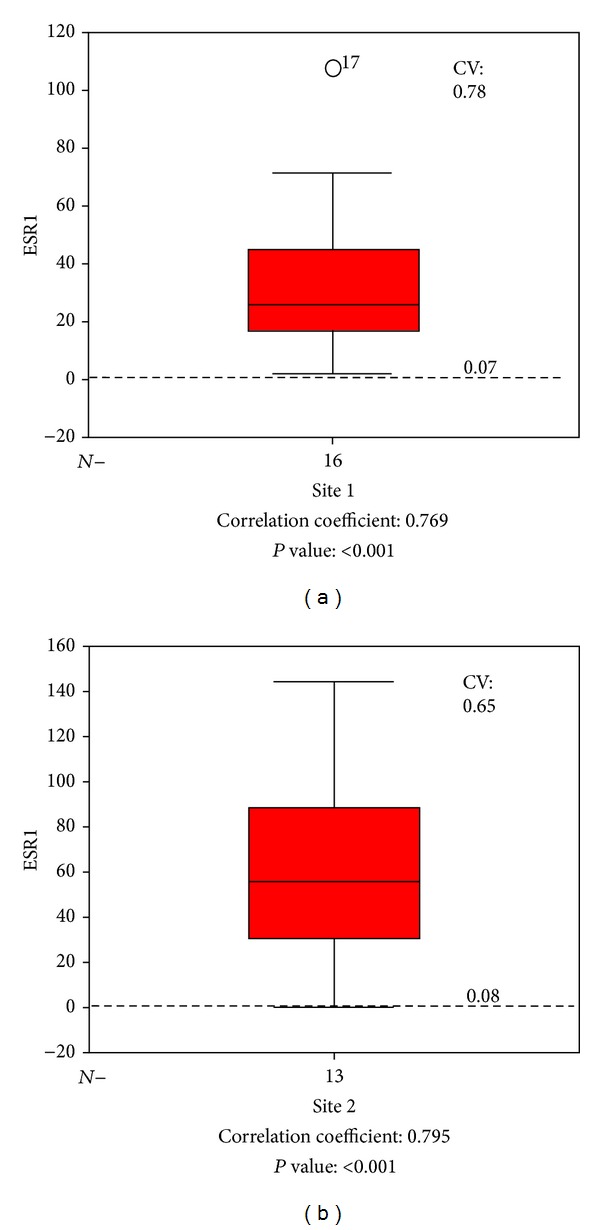
Comparison of the fixation and paraffin embedding procedure.

To establish the influence of tissue fixation and paraffin embedding, we evaluated the correlation between the IHC results with ER gene expression results. For this purpose, we used 35 samples of another study of which IHC results and gene expression results for ESR1 were provided. Of these samples, the ER-positive samples with an Allred score of more than 5 were selected and were used to calculate the coefficients of variation (CV), which was 0.85. Next, the CV-values of the ESR1 gene expression for site 1 and site 2 were calculated, which were 0.78 and 0.65, respectively (Figure 2). ESR1 gene expression of samples with low Allred scores (i.e., 0: dashed line) was significantly lower (i.e., 0.07 and 0.08 for site 1 and site 2, resp.) than those with high (i.e., >5) scores. Statistical analysis showed a significant positive correlation between the scoring results and qRT-PCR results (*P* < 0.05). The correlation coefficients of site 1 and site 2 were 0.769 and 0.795, respectively. These results suggest that formalin fixation and paraffin embedding did not influence the results between different laboratories.

### 3.2. Immunostaining in the Different Laboratories Showed Similar Results

**Figure 3:**
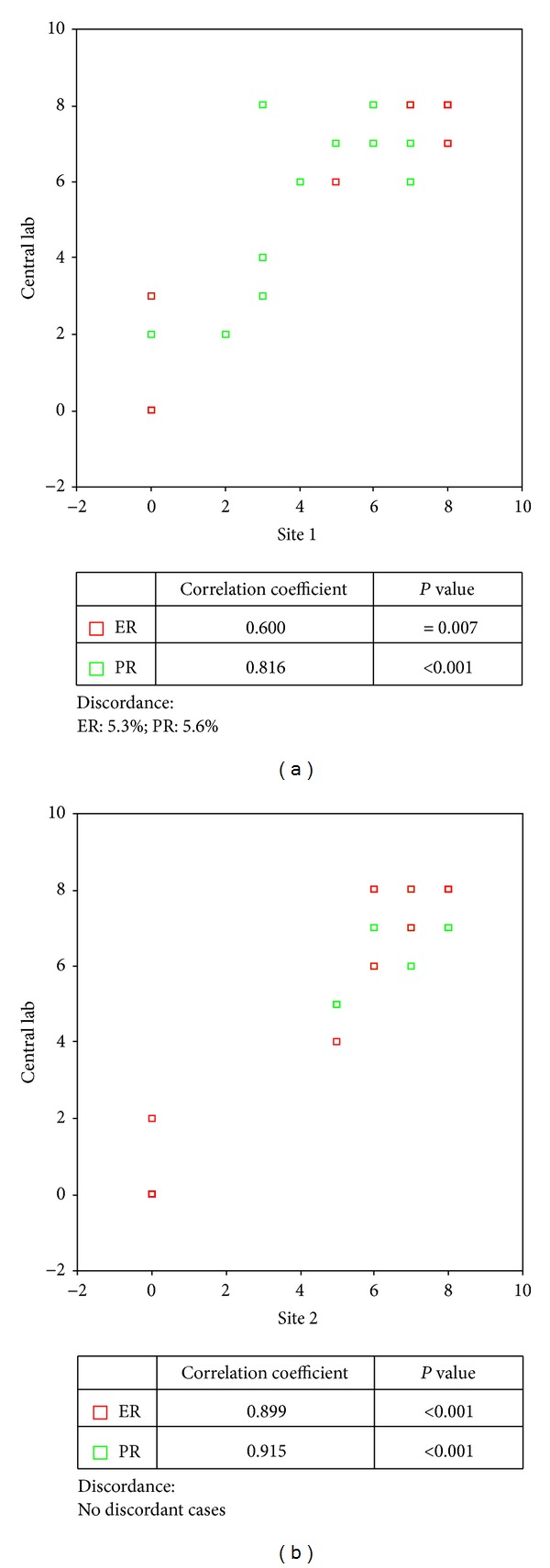
Comparison of the immunostaining method.

In the next step, variability in immunostaining between the peripheral labs and the central lab was evaluated. Slides of 20 tissue samples were stained at the participating laboratories by their proper staining method. Unstained tissue sections from the same tissue block were stained at the central lab using the ER/PR PharmDX kit. All tissue sections were scored blind at the central lab using the Allred scoring system. IHC results between the participating laboratories and the central lab were significantly correlated with a correlation coefficient of higher than 0.600 (*P* < 0.05) (Figure 3). Nevertheless, discordant cases for ER (5.3%) and PR (5.6%) were found for site 1. There were no discordant results between the central lab and site 2.

### 3.3. Scoring Systems of the Different Laboratories Gave Similar Results

**Figure 4:**
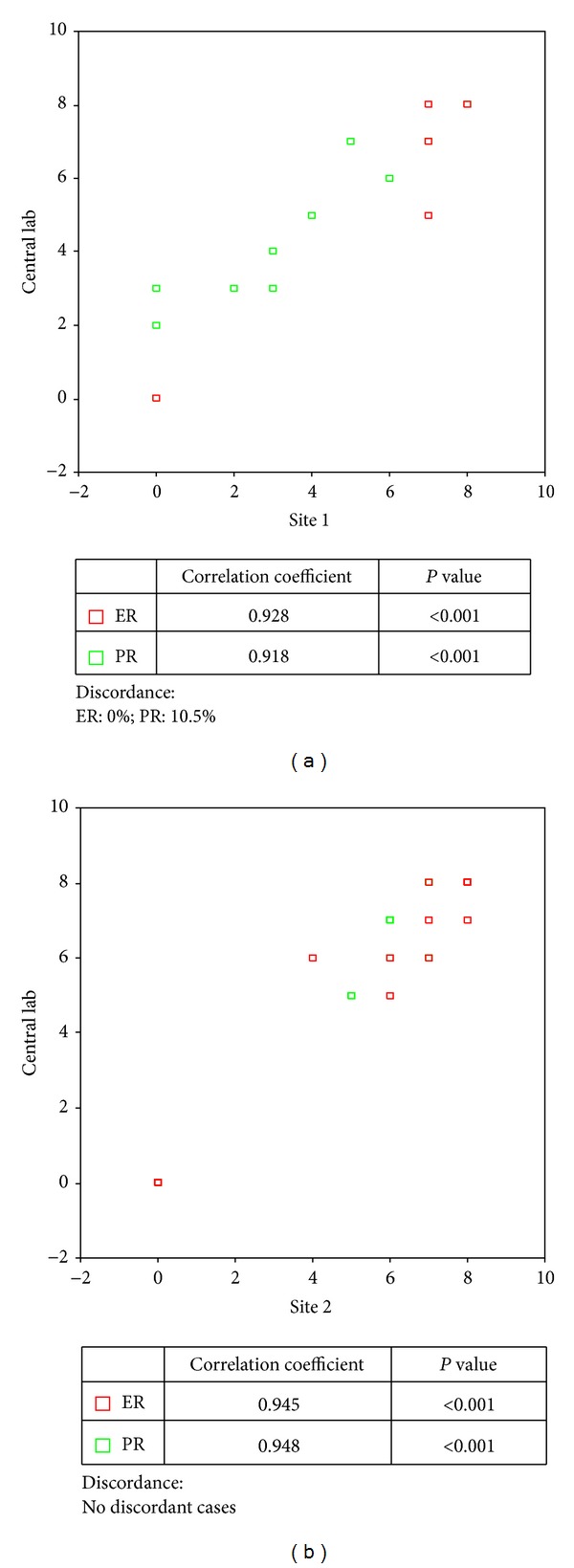
The influence of the scoring system.

Slides stained by the participating laboratories were scored by trained pathologists on site and at the central lab. Comparison of the results of both sites revealed a significant positive correlation of 0.918 and more (*P* < 0.001) (Figure 4). Classifying samples in ER-positive versus ER-negative tumors showed no discordant results. PR scores revealed a discordance of 10.5% for site 1, but no discordant cases for site 2.

### 3.4. ER Pathway Heterogeneity Can Occur in ER-Positive Samples

**Figure 5:**
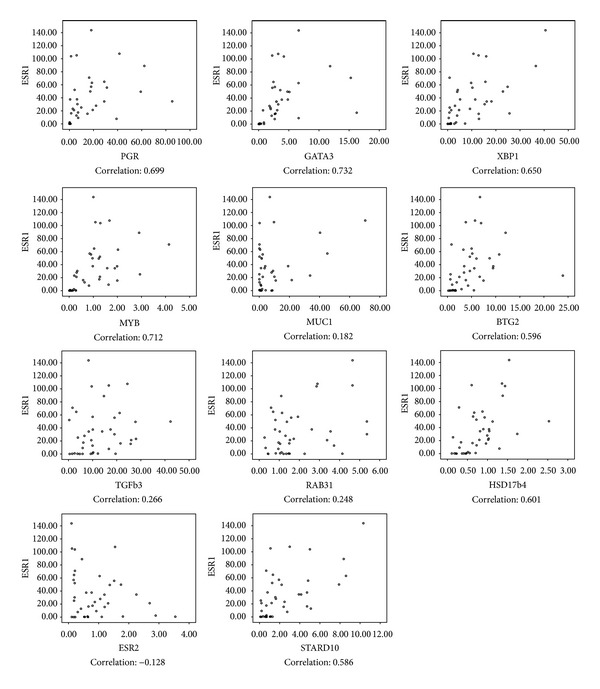
Determination of the pathway activity.

To determine ER pathway activity we performed qRT-PCR for 11 ER target genes on all tissue samples obtained from the participating sites (*N* = 40). The correlations in gene expression between ESR1 and those of the different ER target genes were examined. There was no significant correlation between ESR1 and the following genes: MUC1, TGFb3, RAB31, and ESR2 (Figure 5; Table 1). So the majority of the target genes of ESR1 were correlated with ER-activity.

**Figure 6:**
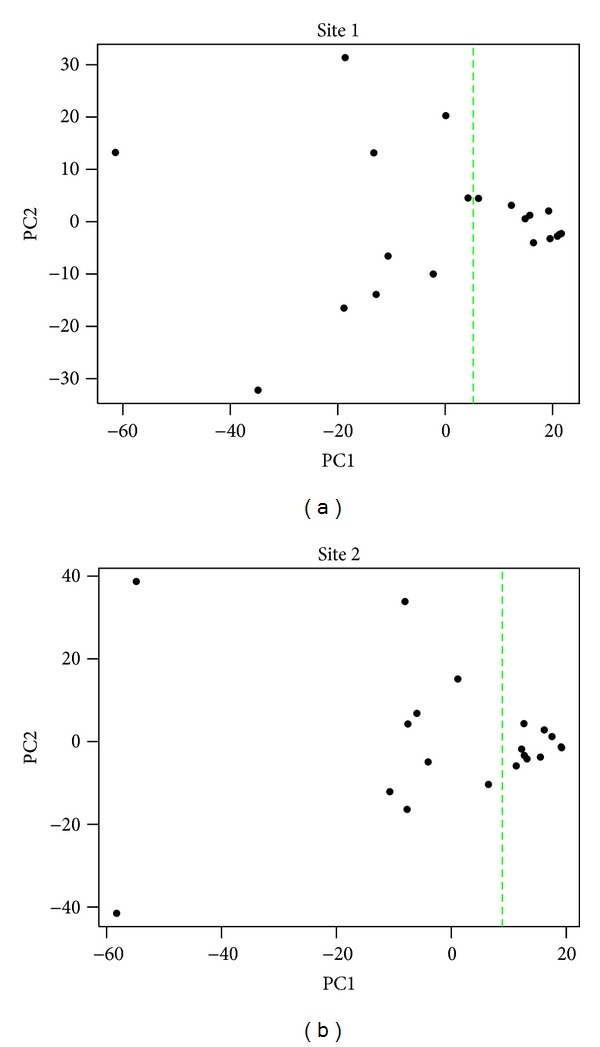
Principal component analysis. Samples were dichotomized relative to the median expression of principal component 1 (green dashed line). For site 1 all samples had comparable ER pathway activation. For site 2, the ER pathway was differential activated in the different samples.

Next, a principal component analysis was performed on the ER target gene expression data for each participating site (Figure 6). As principal component 1 (i.e., the *x*-axis) defines the largest variation in ER target gene expression, the samples were dichotomized relative to the median expression of principal component 1 (green dashed line). Next we investigated if ER target genes were differentially expressed between samples segregated along the *x*-axis. For site 1 no ER target genes were differentially expressed indicating no difference in ER pathway activation between the samples to the left and the right of the green dashed line. These results were corroborated when analyzing ER protein expression data. For site 2, ~60% of the ER target genes were differentially expressed between the samples to the left and the right of the green dashed line, indicating ER pathway heterogeneity. Again, these results were corroborated by ER protein expression, as the Allred scores to the left of dashed line were significantly lower as compared to their counterparts (*P* < 0.05).

## 4. Discussion

IHC is a common technique to determine ER and PR status in tumors. In their review, Brouckaert and colleagues describe the importance of (semi)quantification of steroid hormone receptor expression in estimating the benefits of different treatments as chemotherapy and endocrine therapy [8]. As IHC on sections of FFPE tissue is a routine technique used to classify breast cancer in ER-positive versus -negative tumors, it is important to consider that intra- and interlaboratory variabilities exist. Quality assessment studies in the UK and EU showed significant interlaboratory variability, especially for tumors with low ER levels [5, 9]. In our study we saw a positive correlation between the IHC results of the participating laboratories and those of the central lab. But comparing the results awarded to immunostaining and scoring showed some discordant cases.

Fixation, the first step of IHC procedure, could already influence the IHC results. Delay in fixation, pH, underfixation, and overfixation could reduce immunostaining [10–16]. Williams et al. showed that a low pH could affect the morphology of the tissue but may result in a good immunoreactivity [11]. Also the fixation time is important. A minimal fixation time of 6 to 8 hrs was necessary to receive reliable results [12]. Overfixation was shown to be less important as tissue blocks fixed up to 72 hrs and 96 hrs did not show a reduction in ER, PR, and HER2 status [13, 14]. Fixation for more than 57 days could reduce immunostaining [15, 16]. As such long fixation times are not clinically relevant, overfixation would not be a concern in routine pathology labs. In our study, we found no influence of fixation on the results of IHC, as the results of ER-status obtained by IHC were significantly correlated with the qRT-PCR results of the ESR1 gene.

A second step that can differ between laboratories is the immunostaining procedure. The sensitivity of the antigen can have an impact on the results [17–20]. In a study by Cheang and colleagues two ER antibodies, the monoclonal rabbit antibody SP1 and the mouse monoclonal antibody 1D5, were compared. Of the 4105 samples SP1 detected ER positivity in 69.5% of the samples, while 1D5 detected ER positivity in 63.1% of the cases. SP1 showed to be more sensitive than 1D5 [18]. Similar results were found in a study by Huang showing an 8 times higher affinity of SP1 compared to 1D5 [20]. For this reason, it is plausible to use a cocktail of different antibodies to increase the sensitivity [21]. In our study, we used a cocktail of two mouse monoclonal antibodies to ER, 1D5, and ER-2-123, which bind to different regions of the protein. There was a significant positive correlation between the IHC results of tumor slides stained at the central lab and those stained at the participating laboratories. Nevertheless, the correlation for ER was much lower for site 1 in comparison to site 2. Classifying ER-positive versus ER-negative tumors revealed approximately 5% discordant results for ER and PR for site 1, but not for site 2.

Finally, different scoring systems can result in discordant cases between laboratories [22]. Some scoring procedures are based on the evaluation of the proportion of stained cells, while other scoring systems, like the Allred scoring procedure, rely on the evaluation of the proportion of stained cells and the intensity of the staining [23]. Discordant results obtained by one scoring system could result in concordant cases when using the other scoring system [23]. Beside different scoring systems interobserver variability can also occur. Tumors with low levels of ER expression are often difficult to classify and can lead to observer discordance [24]. In our study the scoring results of the central lab using the Allred scoring method and those of the participating lab using their proper scoring system were significantly positive correlated for ER and PR. Again, despite the high concordance, discordant cases occurred.

As there were some discordant cases between different labs using IHC, it may be interesting to use a more robust, observer-independent technique. Quantitative mRNA-based measurements could be a suitable method to analyze the expression of ESR1 and PR. Studies comparing microarray mRNA quantification and qRT-PCR with IHC revealed a high concordance [25–30].

These results confirm that qRT-PCR could be an additional method for determining the status of hormonal receptors. Besides the advantage of being observer independent, qRT-PCR also allows to detect heterogeneity in the activity of the ER-signalling pathway of the different tumors as shown in our study. The assessment of ER-activity is of importance because not all patients with ER-positive breast cancer do respond to endocrine therapy [31].

## 5. Conclusions

IHC is a routinely used procedure to classify breast cancer in ER-positive and -negative tumors. Despite good overall concordance, discordant cases determined by preanalytical and analytical lab-specific procedures have been identified. These discordant cases lead to misclassification of tumors. qRT-PCR could be an additional observer-independent technique to determine hormone receptor status and activity. The weakness of the study is the low number of samples. It would also be interesting to use more samples with low ER and PR expression in the study.

## Figures and Tables

**Table 1:** Determination of the pathway activity of ER.

Gene	Site 1		Site 2	
	Correlation with ESR1	*P* value	Correlation with ESR1	*P* value
PGR	0.588	0.006	0.693	0.001
GATA3	0.582	0.007	0.798	0.000
XBP1	0.561	0.010	0.773	0.000
MYB	0.573	0.008	0.901	0.000
MUC1	0.186	0.431	0.819	0.424
BTG2	0.472	0.036	0.701	0.001
TGFb3	0.072	0.762	0.329	0.156
RAB31	0.206	0.384	0.302	0.195
HSD17b4	0.498	0.026	0.668	0.001
ESR2	−0.137	0.565	−0.203	0.391
STARD10	0.447	0.048	0.714	0.000
